# Pan‐Cancer Survival Impact of Immune Checkpoint Inhibitors in a National Healthcare System

**DOI:** 10.1002/cam4.70379

**Published:** 2024-11-07

**Authors:** Sean R. Miller, Matthew Schipper, Lars G. Fritsche, Ralph Jiang, Garth Strohbehn, Erkin Ötleş, Benjamin H. McMahon, Silvia Crivelli, Rafael Zamora‐Resendiz, Nithya Ramnath, Shinjae Yoo, Xin Dai, Kamya Sankar, Donna M. Edwards, Steven G. Allen, Michael D. Green, Alex K. Bryant

**Affiliations:** ^1^ Department of Radiation Oncology Veterans Affairs Ann Arbor Healthcare System Ann Arbor Michigan USA; ^2^ Department of Radiation Oncology University of Michigan Ann Arbor Michigan USA; ^3^ Department of Biostatistics University of Michigan Ann Arbor Michigan USA; ^4^ Center for Statistical Genetics University of Michigan Ann Arbor Michigan USA; ^5^ Veterans Affairs Center for Clinical Management Research Ann Arbor Michigan USA; ^6^ Division of Medical Oncology, Department of Medicine Veterans Affairs Ann Arbor Healthcare System Ann Arbor Michigan USA; ^7^ Division of Hematology/Oncology, Department of Medicine University of Michigan Ann Arbor Michigan USA; ^8^ Rogel Cancer Center University of Michigan Ann Arbor Michigan USA; ^9^ Medical Scientist Training Program University of Michigan Medical School Ann Arbor Michigan USA; ^10^ Theoretical Biology and Biophysics Los Alamos National Laboratory Los Alamos New Mexico USA; ^11^ Applied Mathematics and Computational Research Division Lawrence Berkeley National Laboratory Berkeley California USA; ^12^ Computational Science Initiative Brookhaven National Laboratory Upton New York USA; ^13^ Division of Medical Oncology, Department of Medicine Samuel Oschin Comprehensive Cancer Institute, Cedars‐Sinai Medical Center Los Angeles California USA

**Keywords:** check point control, clinical cancer research, clinical observations, immune checkpoint inhibitors

## Abstract

**Background:**

The cumulative, health system‐wide survival benefit of immune checkpoint inhibitors (ICIs) is unclear, particularly among real‐world patients with limited life expectancies and among subgroups poorly represented on clinical trials. We sought to determine the health system‐wide survival impact of ICIs.

**Methods:**

We identified all patients receiving PD‐1/PD‐L1 or CTLA‐4 inhibitors from 2010 to 2023 in the national Veterans Health Administration (VHA) system (ICI cohort) and all patients who received non‐ICI systemic therapy in the years before ICI approval (historical control). ICI and historical control cohorts were matched on multiple cancer‐related prognostic factors, comorbidities, and demographics. The effect of ICI on overall survival was quantified with Cox regression incorporating matching weights. Cumulative life‐years gained system‐wide were calculated from the difference in adjusted 5‐year restricted mean survival times.

**Results:**

There were 27,322 patients in the ICI cohort and 69,801 patients in the historical control cohort. Among ICI patients, the most common cancer types were NSCLC (46%) and melanoma (10%). ICI demonstrated a large OS benefit in most cancer types with heterogeneity across cancer types (NSCLC: adjusted HR [aHR] 0.56, 95% confidence interval [CI] 0.54–0.58, *p* < 0.001; urothelial: aHR 0.91, 95% CI 0.83–1.01, *p* = 0.066). The relative benefit of ICI was stable across patient age, comorbidity, and self‐reported race subgroups. Across VHA, 15,859 life‐years gained were attributable to ICI within 5‐years of treatment, with NSCLC contributing the most life‐years gained.

**Conclusion:**

We demonstrated substantial increase in survival due to ICIs across a national health system, including in patient subgroups poorly represented on clinical trials.

## Graphical abstract

In this matched historical cohort study in the Veterans Health Administration system, ICIs had a heterogenous survival benefit by cancer type, and poorer‐prognosis patients derived smaller absolute survival benefits. System‐wide, ICIs accounted for nearly 16,000 life‐years gained within a 5‐year time horizon after treatment.

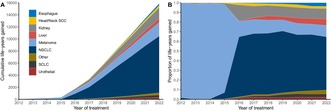

## 1 Introduction

Since the FDA approval of ipilimumab in 2011 for melanoma, indications for immune checkpoint inhibitors (ICI) have rapidly expanded to over 20 cancer types [1, 2, 3, 4, 5, 6]. While ICIs were quickly adopted after practice‐changing clinical trials [7], many of which showed an overall survival benefit, real‐world patients are older, have more comorbidities, and are more likely to belong to marginalized populations than clinical trial patients [8]. Alarmingly, enrollment of Black patients on ICI trials has declined over the last decade [9]. ICIs carry the risk of significant side effects, time toxicity, and financial toxicity, and are partly responsible for significant increases in spending on cancer care across health systems [10, 11]. It is, therefore, essential to examine the implementation of these drugs and confirm their efficacy in diverse populations and with adequate statistical power to examine rare patient subgroups and multiple cancer types.

As the largest integrated health system in the USA and as a single‐payer system serving a diverse population, the Veterans Health Administration (VHA) is uniquely positioned to provide insight into national prescribing trends as well as long‐term outcomes [12]. In this study, we sought to describe the system‐wide survival impact of ICIs relative to historical survival outcomes. We further sought to describe heterogeneity of real‐world ICI effectiveness across cancer types and among patient subgroups poorly represented in clinical trials.

## 2 Methods

### 2.1 Data Source

This study used electronic medical record data from the VHA Corporate Data Warehouse (CDW), which includes data for all Veterans receiving care through VHA facilities nationwide. This study was approved by the Veterans Affairs Ann Arbor institutional review board. Health Insurance Portability and Accountability Act authorization was waived because the study analyzed retrospective data and involved minimal risk to participants.

### 2.2 ICI Cohort Definition

We queried CDW for all patients who received an ICI (pembrolizumab, nivolumab, atezolizumab, avelumab, durvalumab, tremelilumab, dostarlimab, cemiplimab‐rwlc, ipilimumab, or nivolumab/relatlimab‐rmbw) from January 2010 to August 2023 using CPT codes (Table S1). The date of the first ICI infusion was defined as the index date for each patient. We assigned cancer type and date of diagnosis with a combination of VA Cancer Registry System data and ICD‐9/10 codes; see Methods S1 for details. Endometrial cancer and colorectal cancer were excluded, as the primary indications for ICI in these sites are defined by microsatellite instability‐high (MSI‐H) molecular status (as of the time of manuscript preparation). As MSI‐H status was not available, a comparable historical control cohort could not be constructed for these patients.

### 2.3 Historical Control Cohort

We developed a historical control cohort of patients with the same cancer types as the ICI cohort who were treated with conventional chemotherapeutics or targeted therapies and were never exposed to ICI (see Table S2 and Methods S1 for list of included therapeutics). Patients were included if the systemic therapy start date was within 10 years before the first FDA approval of ICI in each cancer type. We included additional control patients to provide matches for patients with adjuvant ICI indications (Methods S1 and Table S3). We excluded patients undergoing systemic therapy regimens that were not concordant with their cancer type (*n* = 7454, 9%), defined using National Comprehensive Cancer Network treatment guidelines (accessed 8/14/23) to identify individual drugs or combination regimens that have been used in each cancer type.

### 2.4 Time Trends in ICI Uptake

We performed descriptive analyses of the absolute number and percentage of patients with each cancer type in the national VA system who received any ICI during their care. To allow for stratification by stage at diagnosis, we limited the cohort to patients in the VA cancer registry data. The total number of patients diagnosed with each cancer type was quantified from 2010 to 2021. The number and proportion of patients who received ICI during their subsequent care were then quantified by year and cancer type.

### 2.5 Covariates

We captured demographic information from CDW including self‐reported race (White, Black, or other), ethnicity (Hispanic vs. non‐Hispanic), marital status (married, divorced, never married, or other), and sex (male or female). Cancer stage at diagnosis was derived from the Cancer Registry System if available. To capture extent and duration of prior treatment, we quantified the number of unique systemic therapy agents from the date of diagnosis to index date, the number of prior lines of systemic therapy, prior radiation therapy (defined by CPT codes for radiation delivery and/or Cancer Registry System records of radiation therapy), and the elapsed time from cancer diagnosis to the index date. The date of initial cancer diagnosis was defined as the date of diagnosis in the Cancer Registry System data or, if not available, the first appearance of the cancer type ICD code in the patient's data within the 5 years prior to the index date. Charlson Comorbidity Index (CCI) based on ICD‐9/10 codes in the year prior to the index date was also calculated; codes for malignancies were excluded from the CCI calculation. Body mass index was calculated using the closest height and weight measurement to the index date. Geographic region was assigned based on the location of therapy on the index date.

### 2.6 Matching

As the indications for ICIs across disease sites has changed over time with increasing use in earlier lines of therapy, we pursued a matching approach intended to balance the ICI and historical control groups on cancer type, extent and duration of prior therapy, stage at diagnosis, and demographic factors. This was intended to mitigate guaranteed‐time bias from comparing survival between ICI patients treated in later lines of therapy and historical control patients treated in earlier lines. To accomplish this, we performed optimal full matching [13] on cancer type, stage at diagnosis, number of unique prior systemic therapy agents, number of prior lines of systemic therapy, prior radiation therapy, time from diagnosis to the index date, body mass index (BMI), region, age, sex, self‐reported race, ethnicity, and Charlson Comorbidity Index. Propensity scores for matching were estimated using Bayesian additive regression trees [14]. The target estimand was the average treatment effect among the treated (ICI patients). Matching performance was assessed with standardized mean differences. For survival comparisons within cancer types, we performed matching within each cancer type. For subgroup analyses stratified by baseline covariates, we performed matching within each level of the covariate and additionally matched on cancer type.

### 2.7 Expected Life‐Years Gained

To calculate cumulative life‐years gained across the VA system attributable to ICIs, we first calculated the difference in 5‐year restricted mean survival times (RMST) between ICI and historical control groups for each cancer site using weighted Kaplan–Meier curves in the matched samples. We then calculated the expected life‐years gained over a 5‐year time horizon by multiplying the cancer site‐specific difference in RMST by the number of patients in the ICI cohort treated in each year. This represents the expected improvement in mean life‐years attributable to ICI within the first 5 years after treatment, among the population who received ICI [15, 16]. Of note, this method assumes that the absolute RMST improvement from ICI was stable over the study period.

### 2.8 Statistical Analysis

Baseline characteristics were compared across cohorts using the t‐test (for continuous variables) or the chi‐square test (for categorical variables). Survival analysis was performed with univariable Cox regression incorporating the matching weights with cluster‐robust standard errors. As ICIs may be associated with non‐proportional hazards relative to standard therapy in some cancer types, we additionally report median survival and 5‐year RMST differences across ICI and historical control groups. The proportional hazards assumption was checked with visual inspection of scaled Schoenfeld residuals. Overall survival was defined as the time from the index date to death from any cause. Patients were censored at the date of last follow‐up (current through 8/14/23). Date of death was obtained from the internal VA death registry. Analysis was performed with R v4.3.1 (R Core Team, Vienna, Austria).

## 3 Results

### 3.1 Cohort Characteristics

The ICI cohort included 27,322 patients and the historical control cohort included 69,801 patients (Table 1). Among ICI patients, the most common cancer type was NSCLC (46%) followed by melanoma (10%), liver (8.0%), and kidney (7.5%). 95% of patients received PD‐1 or PD‐L1 inhibitors, and most patients received ICI alone (72%) without other concurrent chemotherapy or targeted therapies. In comparison to historical control patients, ICI patients were older (70 or older: 53.9% versus 31.5%, *p* < 0.001), more likely to be treated in the second or later line (48% versus 26%, *p* < 0.001), and more likely to be treated greater than 6 months after the diagnosis date (52% versus 33%, *p* < 0.001). In both cohorts, patients were primarily male (95%–97%), of White self‐reported race (74%–76%), and not Hispanic (96%). Median follow‐up among censored patients was 1.5 years in the ICI group and 9.1 years in the historical control group.

**TABLE 1:** Characteristics of the sample.

Characteristic	Historical control, *N* = 69,801	ICI, *N* = 27,322	*p*
Age group (years)			**< 0.001**
59 or less	15,904 (23%)	2616 (9.6%)	
60 to 69	32,180 (46%)	9893 (36%)	
70 to 79	15,798 (23%)	12,096 (44%)	
80 or higher	5919 (8.5%)	2717 (9.9%)	
Marital status			**< 0.001**
Married	32,521 (47%)	13,290 (49%)	
Divorced	21,422 (31%)	8072 (30%)	
Other	9099 (13%)	3031 (11%)	
Never married	6759 (9.7%)	2929 (11%)	
Sex			**< 0.001**
Male	66,231 (95%)	26,450 (97%)	
Female	3570 (5.1%)	872 (3.2%)	
Self‐reported race			**< 0.001**
White	51,380 (74%)	20,721 (76%)	
Black	11,334 (16%)	4619 (17%)	
Other	7087 (10%)	1982 (7.3%)	
Self‐reported ethnicity			**0.010**
Not Hispanic	67,129 (96%)	26,179 (96%)	
Hispanic	2672 (3.8%)	1143 (4.2%)	
BMI group			**< 0.001**
Healthy weight	27,191 (39%)	10,036 (37%)	
Obese	15,863 (23%)	6764 (25%)	
Overweight	22,159 (32%)	8855 (32%)	
Underweight	4588 (6.6%)	1667 (6.1%)	
Region			**< 0.001**
Midwest	15,801 (23%)	7269 (27%)	
North Atlantic	15,416 (22%)	6121 (22%)	
Southeast	14,599 (21%)	5352 (20%)	
Continental	13,670 (20%)	4132 (15%)	
Pacific	10,315 (15%)	4448 (16%)	
Year of diagnosis			**< 0.001**
2001–2005	2159 (3.1%)	—	
2006–2010	25,246 (36%)	—	
2011–2015	35,174 (50%)	378 (1.4%)	
2016–2020	7107 (10%)	15,695 (57%)	
2021–2023	115 (0.2%)	11,249 (41%)	
Cancer type			**< 0.001**
NSCLC	26,680 (38%)	12,500 (46%)	
Head/Neck SCC	10,007 (14%)	1882 (6.9%)	
Liver	6610 (9.5%)	2174 (8.0%)	
SCLC	6279 (9.0%)	1832 (6.7%)	
Kidney	3628 (5.2%)	2040 (7.5%)	
Urothelial	3667 (5.3%)	1971 (7.2%)	
Esophagus	4243 (6.1%)	851 (3.1%)	
Melanoma	1841 (2.6%)	2746 (10%)	
Gastric	2362 (3.4%)	410 (1.5%)	
Breast	2492 (3.6%)	125 (0.5%)	
Lymphoma	1570 (2.2%)	112 (0.4%)	
Cutaneous SCC	277 (0.4%)	501 (1.8%)	
Merkel Cell	110 (0.2%)	173 (0.6%)	
Cervix	35 (< 0.1%)	< 10 (< 0.1%)	
ICI class			—
PD‐1/PD‐L1	—	25,882 (95%)	
Combination (PD‐1/PD‐L1 and CTLA‐4)	—	1060 (3.9%)	
CTLA‐4 alone	—	379 (1.4%)	
ICI regimen type			—
Monotherapy	—	19,697 (72%)	
Combination with other therapy	—	7625 (28%)	
Stage at diagnosis			**< 0.001**
I	6063 (8.7%)	2583 (9.5%)	
II	8280 (12%)	2210 (8.1%)	
III	15,919 (23%)	4901 (18%)	
IV	22,891 (33%)	7200 (26%)	
Unknown	16,648 (24%)	10,428 (38%)	
Cancer type source			**< 0.001**
Cancer registry data	57,550 (82%)	20,643 (76%)	
Diagnosis code	12,251 (18%)	6679 (24%)	
**Prior radiotherapy**	26,834 (38%)	9490 (35%)	**< 0.001**
Prior lines of systemic therapy			**< 0.001**
0	51,598 (74%)	14,208 (52%)	
1 to 2	17,286 (25%)	12,627 (46%)	
3 to 4	870 (1.2%)	466 (1.7%)	
5 or more	47 (< 0.1%)	21 (< 0.1%)	
Number of prior systemic therapies			**< 0.001**
0	51,598 (74%)	14,208 (52%)	
1 to 2	14,102 (20%)	10,522 (39%)	
3 to 4	3732 (5.3%)	2300 (8.4%)	
5 or more	369 (0.5%)	292 (1.1%)	
Charlson Comorbidity Index (excluding malignancies)			**< 0.001**
0	29,994 (43%)	8278 (30%)	
1	22,872 (33%)	8876 (32%)	
2 to 3	13,796 (20%)	7886 (29%)	
4 to 6	2774 (4.0%)	2050 (7.5%)	
7 or higher	365 (0.5%)	232 (0.8%)	
Time from diagnosis to index (months)			**< 0.001**
0 to 6	46,459 (67%)	13,221 (48%)	
7 to 12	8908 (13%)	4473 (16%)	
13 to 18	4597 (6.6%)	2713 (9.9%)	
19 to 24	2752 (3.9%)	1812 (6.6%)	

Abbreviations: ICI, immune checkpoint inhibitors; NSCLC, non‐small‐cell lung cancer; SCC, squamous cell carcinoma; SCLC, small‐cell lung cancer; PD‐1, programmed cell death protein 1; PD‐L1, programmed cell death ligand 1.

*Note:* Bold values signifies the all p‐values.

### 3.2 ICI Utilization Trends

**FIGURE 1:**
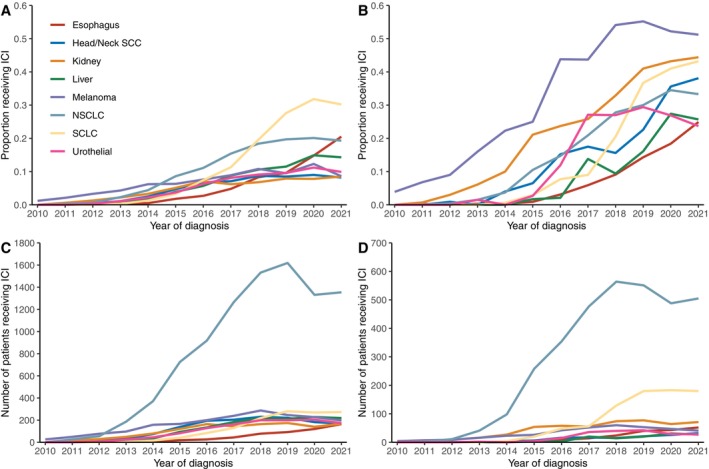
ICI utilization in the national VA system. (A) Proportion of patients receiving ICI, all stages at diagnosis. (B) Proportion of patients receiving ICI, metastatic at diagnosis. (C) Absolute number of patients receiving ICI, all stages at diagnosis. (D) Absolute number of patients receiving ICI, metastatic at diagnosis. ICI: Immune checkpoint inhibitors; SCC: Squamous cell carcinoma; NSCLC: Non‐small‐cell lung cancer; SCLC: Small‐cell lung cancer.

Utilization of ICI increased over time since 2010 for all cancer types (Figure 1A). Among patients with metastatic disease at diagnosis, ICI was most commonly used in melanoma (2021 rate: 51.2%) followed by kidney cancer (44.4%), SCLC (43.0%), and head/neck SCC (39.3%) (Figure 1B). By absolute number of patients, NSCLC was the most common diagnosis among ICI‐treated patients since 2013 and saw the steepest rise in that period (Figure 1C,D). Among patients who received ICI and were metastatic at diagnosis, the use of ICI in the first‐line setting also increased over time (2021 rate of first‐line ICI use: melanoma: 95.1%; NSCLC: 86.4%; SCLC: 73.2%; head/neck SCC: 67%; kidney: 63.4%).

### 3.3 ICI Impact on Overall Survival

**FIGURE 2:**
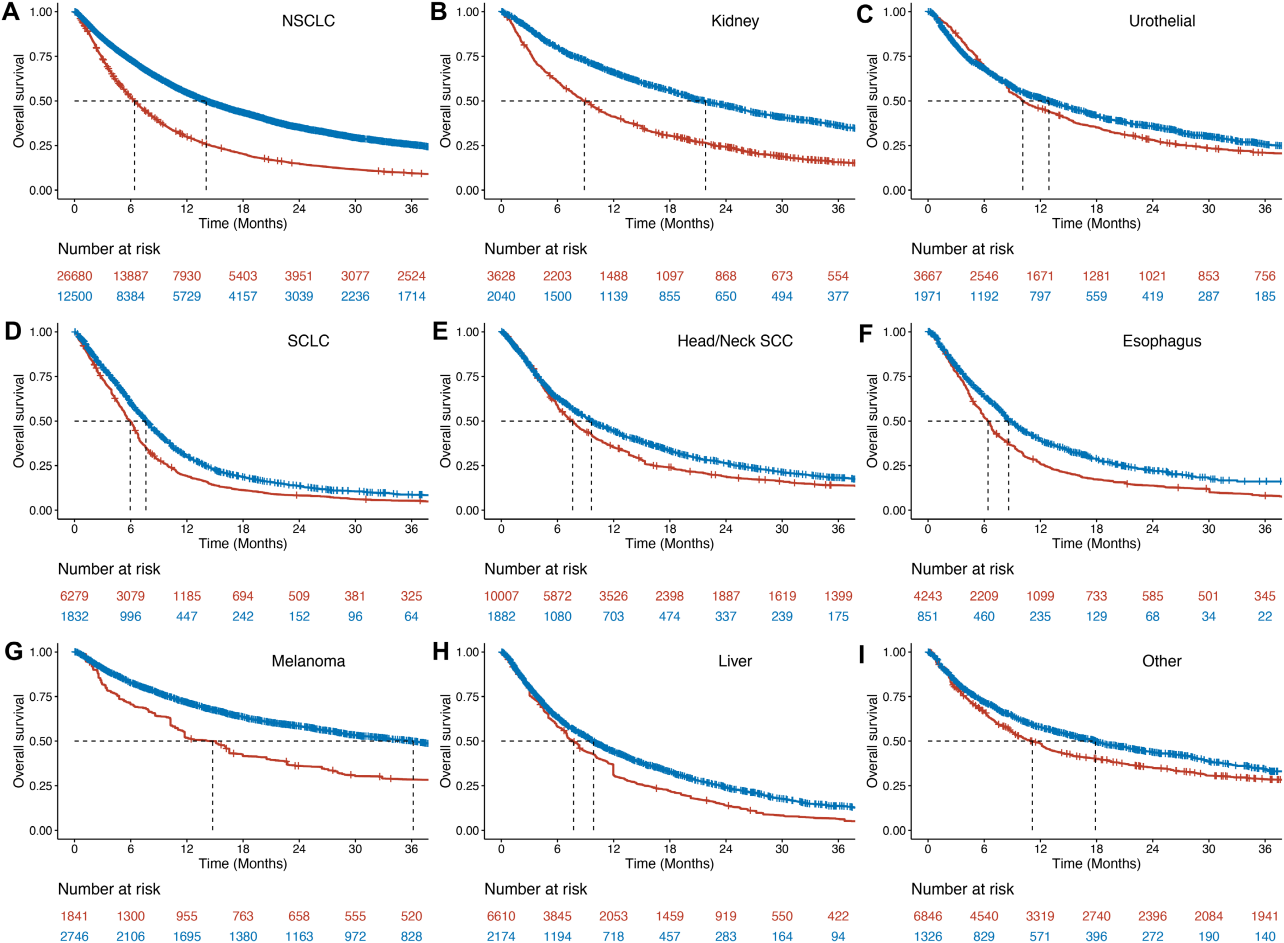
Overall survival by cancer type. Weighted Kaplan–Meier overall survival curves for ICI (blue) and historical control (red) cohorts, by cancer type (panels A‐I). Dotted lines indicated median survival times. ICI, immune checkpoint inhibitors; SCC, squamous cell carcinoma; NSCLC, non‐small‐cell lung cancer; SCLC, small‐cell lung cancer.

After the matching procedure, all cancer types showed excellent matching of ICI and historical control patients; across all cancer types (*n* = 341 matched variables), the absolute standardized difference was < 0.20 in 100% of variables, < 0.10 in 96%, and < 0.05 in 74%. In weighted Cox regression of the matched sample, ICI was significantly associated with a survival benefit in most cancer types except for urothelial cancer (*p* = 0.066; Table 2; Figure 2). The relative magnitude of ICI benefit differed across cancer types, with hazard ratios ranging from 0.56 (NSCLC) to 0.91 (urothelial). The absolute magnitude of median survival improvement similarly differed by cancer type, ranging from 22 months (melanoma; 36.2 months in ICI vs. 14.8 months in historical control) to 1.7 months (SCLC; 7.6 months in ICI vs. 6.9 months in historical control). The difference in 5‐year RMST similarly differed across cancer types, with the largest benefits seen in kidney (11.4 months) and melanoma (10.8 months) and the smallest benefit in urothelial (2.1 months). Of note, the proportional hazards assumption was violated in urothelial, head/neck SCC, and melanoma; in these cancers the relative benefit of ICI increased with follow‐up time.

**TABLE 2:** Overall survival regression results by cancer type and baseline covariate subgroups.

Variable	Level	aHR for ICI (95% CI)	*p*	Median survival (control)	Median survival (ICI)	Median survival difference	RMST (control)	RMST (ICI)	RMST difference
Cancer type	NSCLC	0.56 (0.54–0.58)	< 0.001	6.41	14.06	7.65	12.54	22.46	9.92
	SCLC	0.75 (0.69–0.81)	< 0.001	5.95	7.62	1.67	9.53	12.64	3.11
	Melanoma	0.60 (0.49–0.72)	< 0.001	14.75	36.17	21.42	24.01	34.78	10.77
	Kidney	0.54 (0.49–0.59)	< 0.001	8.87	21.82	12.95	16.73	28.17	11.44
	Head/Neck SCC	0.85 (0.79–0.93)	< 0.001	7.62	9.63	2.01	15.39	18.08	2.69
	Liver	0.75 (0.66–0.84)	< 0.001	7.72	9.86	2.14	11.99	16.38	4.39
	Urothelial	0.91 (0.83–1.01)	0.066	10.12	12.91	2.79	20.01	22.09	2.08
	Esophagus	0.72 (0.63–0.82)	< 0.001	6.41	8.61	2.20	11.87	17.00	5.13
	Other	0.81 (0.72–0.91)	< 0.001	11.14	17.87	6.73	22.84	26.57	3.73
Age group	59 or less	0.67 (0.62–0.73)	< 0.001	8.48	17.68	9.20	19.11	26.62	7.51
	60 to 69	0.64 (0.61–0.68)	< 0.001	7.39	13.90	6.51	15.06	23.07	8.01
	70 to 79	0.62 (0.59–0.66)	< 0.001	7.33	13.44	6.11	14.29	22.53	8.24
	80 or higher	0.74 (0.67–0.83)	< 0.001	7.66	10.97	3.31	13.90	19.03	5.13
Self‐reported race	White	0.67 (0.64–0.69)	< 0.001	7.82	13.50	5.68	15.44	22.78	7.34
	Black	0.62 (0.58–0.66)	< 0.001	7.29	14.29	7.00	14.44	22.77	8.33
Charlson Comorbidity Index	0	0.66 (0.63–0.70)	< 0.001	8.71	17.51	8.80	18.41	26.24	7.83
	1	0.62 (0.59–0.65)	< 0.001	7.03	13.27	6.24	14.01	22.36	8.35
	2 to 3	0.66 (0.62–0.70)	< 0.001	6.93	12.32	5.39	13.74	20.87	7.13
	4 to 6	0.71 (0.63–0.80)	< 0.001	6.80	10.09	3.29	12.72	18.38	5.66
BMI	Healthy weight	0.63 (0.60–0.66)	< 0.001	6.08	11.27	5.19	12.26	20.01	7.75
	Obese	0.68 (0.63–0.73)	< 0.001	10.38	18.69	8.31	19.56	26.71	7.15
	Overweight	0.63 (0.59–0.66)	< 0.001	7.89	15.47	7.58	15.95	24.42	8.47
	Underweight	0.65 (0.59–0.71)	< 0.001	4.37	7.75	3.38	9.13	15.41	6.28
Prior lines of systemic therapy	0	0.69 (0.66–0.72)	< 0.001	8.48	14.92	6.44	17.47	24.31	6.84
	1 to 2	0.61 (0.58–0.64)	< 0.001	7.00	12.88	5.88	13.07	21.55	8.48
	3 to 4	0.70 (0.57–0.86)	< 0.001	6.60	8.97	2.37	11.86	17.69	5.83
Prior radiation therapy	Yes	0.60 (0.56–0.63)	< 0.001	6.57	12.98	6.41	13.02	21.90	8.88
	No	0.68 (0.66–0.71)	< 0.001	8.28	14.16	5.88	16.39	23.37	6.98

*Note:* Survival and RMST are reported in months. RMST is calculated over a 5‐year time horizon.

Abbreviations: aHR, adjusted hazard ratio; BMI, body mass index; CI, confidence interval; ICI, immune checkpoint inhibitors; NSCLC, non‐small‐cell lung cancer; RMST, restricted mean survival time; SCC, squamous cell carcinoma; SCLC, small‐cell lung cancer.

In analyses stratified by baseline covariate subgroups, the adjusted hazard ratio for ICI (versus historical control) was largely stable across age, race, CCI, BMI, number of lines of systemic therapy, and prior radiation treatment (Table 2). However, the absolute benefit of ICI declined in poorer‐prognosis subgroups (median survival benefit 9.2 months for patients 59 or younger vs. 3.3 months for 80 or older; 8.8 months for CCI 0 versus 3.3 months for CCI 4 to 6; 5.2 months for healthy weight vs. 3.4 months for underweight; 6.4 months for 0 prior lines of systemic therapy vs. 2.4 months for 3–4 prior lines of systemic therapy).

### 3.4 ICI Impact on Expected Life‐Years Gained

**FIGURE 3:**
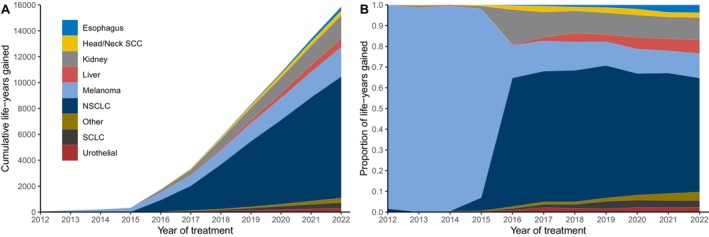
Life‐years gained across the Veterans Affairs system attributable to immune checkpoint inhibitors. (A) Cumulative absolute expected life‐years gained attributable to ICI among patients treated from 2012 to 2022, calculated over a 5‐year time horizon after treatment. (B) Percentage of life‐years gained attributable to each cancer type.

We then used the estimated average improvement in RMST to calculate the cumulative number of life‐years gained attributable to ICI within a 5‐year time horizon after treatment (Figure 3). Among patients treated from 2012 to 2022, we estimate there were 15,859 cumulative life‐years gained across the VA system. NSCLC accounted for 9380 cumulative life‐years gained, followed by melanoma (2265), kidney (1768), and liver (678) (Figure 3A). For patients treated from 2012 to 2015, melanoma accounted for most life‐years gained (96%; Figure 3B). For patients treated in 2016 onward, NSCLC became the dominant contributor both to absolute life‐years gained (Figure 3A) and in percentage terms (Figure 3B). NSCLC accounted for 55% of life‐years gained among patients treated in 2022.

## 4 Discussion

In this pan‐cancer study of ICI uptake and survival outcomes in the United States' largest integrated healthcare system, we demonstrate rapid uptake of ICI across cancer types and a profound population‐level survival benefit. Over a five‐year time horizon after treatment, we estimate almost 16,000 cumulative life‐years gained attributable to ICI system‐wide, predominately from patients with NSCLC. We found wide variation across cancer types in the relative and absolute survival gains from ICI, with some cancer types showing only marginal survival improvements over historical controls. In subgroup analyses, we found that patients in poorer‐prognosis subgroups—older, heavily pre‐treated, highly comorbid, or underweight patients—experience smaller absolute survival benefits. Importantly, we found a robust survival benefit to ICI among Black patients, who have been poorly represented in ICI clinical trials [17]. Taken together, our work confirms the health system‐wide benefits of ICI as a drug class while also highlighting important variation in absolute and relative survival gains.

We found the largest absolute benefits from ICI in patients with melanoma, kidney cancer, and NSCLC, with a magnitude of benefit similar to published RCTs. In NSCLC, ICIs showed an approximately 3 month median overall survival benefit in the second line of therapy for advanced or recurrent disease, 5–9 months in the first line, and 18 months in the adjuvant setting [5, 18, 19, 20, 21, 22, 23, 24, 25, 26]. The median overall survival improvement in our cohort was intermediate to these trials at 7.7 months, likely reflecting the mixed composition of the VA population across disease stages. In melanoma, CheckMate 067 demonstrated a 20.9 month overall survival benefit with combined first‐line ipilimumab and nivolumab compared to ipilimumab alone, which had previously been demonstrated to be superior to dacarbazine or gp100 vaccine in the first line [1, 6, 27]. In the adjuvant setting, comparable relative improvements have been noted in distant metastasis‐free survival relative to placebo [28]. We found a similar magnitude of median overall survival benefit of 21 months, though again comparisons are limited by the mix of disease stages and indications in our real‐world dataset. Modest overall survival benefits were seen in SCLC, head and neck SCC, esophageal cancer, and liver cancer, with observed median overall survival improvements of 2–3 months concordant with published results of RCTs in these cancers [29, 30, 31, 32, 33, 34, 35, 36, 37, 38].

We found a marginal benefit to ICI in urothelial cancer, similar to published randomized literature [39]. Across multiple trials, the maximum improvement in median survival was approximately 7 months in JAVELIN, while other trials have shown modest or no benefit in survival [40, 41, 42]. Interestingly, we found a violation of proportional hazards in urothelial cancer similar to that observed in Keynote 045 [40], with ICI associated with worse survival in the first 4–6 months but improved thereafter. This highlights a need to identify the urothelial cancer patients experiencing early mortality events after ICI initiation and understand the underlying causes.

This heterogeneity in ICI benefit across cancer types was further reflected in our subgroup analyses, where we found lower absolute survival improvements in poorer‐prognosis patient subgroups. While the absolute benefit of ICI declined with poorer prognosis, the relative benefit was largely stable with a hazard ratio of approximately 0.65 across subgroups. The variation in absolute benefit suggests that the absolute survival benefit should be carefully weighed against the risk of immune‐related toxicities and the side effects of alternative treatments when prescribing ICIs to patients with poor prognosis who were not well‐represented on ICI clinical trials. On the other hand, we showed a reassuringly large survival benefit of ICI among Black patients, a group for whom there is a paucity of ICI effectiveness data [17].

On a population level, we estimate that ICI use has generated almost 16,000 life‐years gained in the first 5 years after treatment since 2011. Until 2015, the majority of life‐years gained were attributable to melanoma, the first cancer type in which ICI drugs were approved [1]. After 2015, the majority of life‐years gained from ICI have been among NSCLC patients following the introduction of ICIs in advanced or recurrent NSCLC. The dominance of NSCLC is driven by the high incidence of NSCLC among Veterans (in part due to higher rate of smoking and occupational exposures) [43, 44], the responsiveness of NSCLC to ICIs, and the large relative and absolute survival benefit observed in clinical trials. While we also observed large absolute life‐year gains in melanoma and kidney cancer, these disease sites were relatively rare and, therefore, had less of a system‐wide impact.

Our study has several limitations. Despite multiple methods of ascertainment, we were unable to ascribe cancer type in 5% of patients. Due to the lack of Cancer Registry System data for all patients, stage at diagnosis was missing in ~25% of patients. We had limited ability to adjust for cancer‐related characteristics at time of treatment (e.g., anatomic distribution of disease, number and size of measurable tumors, PD‐L1, tumor mutational burden) due to a lack of structured cancer‐related information at the time of treatment. As our estimates of life‐years gained were calculated over a 5‐year time horizon, our methodology will underestimate the benefits for long‐term survivors, for whom the benefit of ICI will continue to accrue over their remaining lifespan. While we demonstrate clear survival benefits to ICI, we do not consider the financial costs and toxicities of prolonged ICI courses to health systems, patients, and caregivers. The cost‐effectiveness of ICI remains unclear in cancer types with marginal survival benefits and among poor‐prognosis patient subgroups [45]. Alternative methods of dosing [46, 47] and shorter treatment durations [48] could improve the cost‐effectiveness and reduce the logistical demands imposed by this critically important class of drugs. Finally, the Veteran population is primarily male, limiting our ability to estimate the effect of ICI in female‐predominant cancer types. The Veteran population may not be representative of the general population in terms of comorbidity, cancer incidence, and socioeconomics, which could further limit generalizability of our results [49].

In conclusion, we demonstrate rapid uptake of ICIs and large survival benefits on a health system‐wide scale, attributable in large part to the efficacy of ICI in NSCLC and high prevalence of this cancer in the Veteran population. However, we identify important variation in real‐world effectiveness among patient subgroups and across cancer types, reflective of the varying strength of randomized evidence supporting ICI efficacy in different cancers and the heterogeneity of real‐world patient populations.

## Author Contributions

**Sean R. Miller:** conceptualization (equal), formal analysis (equal), investigation (equal), methodology (equal), writing – original draft (lead), writing – review and editing (equal). **Matthew Schipper:** conceptualization (supporting), formal analysis (equal), writing – review and editing (supporting). **Lars G. Fritsche:** conceptualization (supporting), writing – review and editing (supporting). **Ralph Jiang:** conceptualization (equal), formal analysis (equal), writing – review and editing (supporting). **Garth Strohbehn:** writing – review and editing (equal). **Erkin Ötleş:** writing – review and editing (equal). **Benjamin H. McMahon:** conceptualization (equal), writing – review and editing (equal). **Silvia Crivelli:** conceptualization (equal), writing – review and editing (equal). **Rafael Zamora‐Resendiz:** conceptualization (equal), writing – review and editing (equal). **Nithya Ramnath:** conceptualization (equal), writing – review and editing (equal). **Shinjae Yoo:** conceptualization (equal), writing – review and editing (equal). **Xin Dai:** conceptualization (equal), writing – review and editing (equal). **Kamya Sankar:** writing – review and editing (equal). **Donna M. Edwards:** conceptualization (equal), writing – review and editing (equal). **Steven G. Allen:** conceptualization (supporting), writing – review and editing (equal). **Michael D. Green:** conceptualization (equal), project administration (equal), supervision (supporting), writing – review and editing (equal). **Alex K. Bryant:** conceptualization (lead), data curation (lead), formal analysis (lead), investigation (lead), methodology (lead), project administration (lead), resources (lead), software (lead), supervision (lead), validation (lead), visualization (lead), writing – review and editing (lead).

## Conflicts of Interest

The authors report no conflicts of interest.

## Supporting information


Data S1.

## Data Availability

Data are unable to be released due to HIPAA and Veterans Affairs policy, however all data are available to approved Veterans Affairs researchers.
