# Supplementary material for: Pan‐Cancer Survival Impact of Immune Checkpoint Inhibitors in a National Healthcare System
Source: Cancer Med. 2024 Nov 7;13(21):e70379. doi: 10.1002/cam4.70379 (PMC11541111; doi:10.1002/cam4.70379)
Supplement: Supplementary file 1 — Data S1. [file CAM4-13-e70379-s001.docx]

**Supplemental Material for:**

Pan-cancer survival impact of immune checkpoint inhibitors in a national healthcare system.

Sean R. Miller, MD;^1,2^ Matthew Schipper, PhD;^3^ Lars G. Fritsche, PhD;^3,4^ Ralph Jiang, MS;^3^ Garth Strohbehn, MD, MPhil;^5,6,7,8^ Erkin Ötleş, MS;^9^ Benjamin H. McMahon, PhD;^10^ Silvia Crivelli, PhD;^11^ Rafael Zamora-Resendiz, BS;^11^ Nithya Ramnath, MBBS;^6,7^ Shinjae Yoo, PhD;^12^ Xin Dai, PhD;^12^ Kamya Sankar, MD;^13^ Donna M. Edwards, MD, PhD;^1,2^ Steven G. Allen, MD, PhD^1,2^ Michael D. Green, MD, PhD*^1,2^ Alex K. Bryant, MD, MAS*^1,2^

^1^Department of Radiation Oncology, Veterans Affairs Ann Arbor Healthcare System, Ann Arbor, Michigan; ^2^Department of Radiation Oncology, University of Michigan, Ann Arbor, Michigan; ^3^Department of Biostatistics, University of Michigan, Ann Arbor, Michigan;

^4^Center for Statistical Genetics, University of Michigan, Ann Arbor, Michigan; ^5^Veterans Affairs Center for Clinical Management Research, Ann Arbor, Michigan; ^6^Division of Medical Oncology, Department of Medicine, Veterans Affairs Ann Arbor Healthcare System, Ann Arbor, Michigan; ^7^Division of Hematology/Oncology, Department of Medicine, University of Michigan, Ann Arbor, Michigan; ^8^Rogel Cancer Center, University of Michigan, Ann Arbor, Michigan; ^9^Medical Scientist Training Program, University of Michigan Medical School, Ann Arbor, Michigan; ^10^Theoretical Biology and Biophysics, Los Alamos National Laboratory, Los Alamos, New Mexico; ^11^Applied Mathematics and Computational Research Division, Lawrence Berkeley National Laboratory, Berkeley, California; ^12^Computational Science Initiative, Brookhaven National Laboratory, Upton, New York; ^13^Division of Medical Oncology, Department of Medicine, Samuel Oschin Comprehensive Cancer Institute, Cedars-Sinai Medical Center, Los Angeles, California.

*These authors contributed equally.

**Correspondence:**

Alex K. Bryant, MD, MAS

Department of Radiation Oncology

Veterans Affairs Ann Arbor Healthcare System

2215 Fuller Rd., Ann Arbor, Michigan 48105

[bralex@med.umich.edu](mailto:bralex@med.umich.edu)

(734) 845-3914

**Supplemental Methods.**

*Assignment of cancer type.*

To assign cancer type to each ICI patient, we first queried the VA Cancer Registry System data for all diagnoses of a cancer type with an approved ICI indication (as of 8/26/23) and with date of diagnosis within 5 years before the index date. Queried cancer types included non-small-cell lung cancer, small-cell lung cancer, cutaneous melanoma, clear-cell renal cell carcinoma, urothelial carcinoma of the renal pelvis, ureter, or bladder, hepatocellular carcinoma, esophageal or gastroesophageal junction cancer, gastric cancer, squamous cell carcinoma of the head and neck, Hodgkin lymphoma, primary mediastinal B-cell lymphoma, cutaneous squamous cell carcinoma, Merkel cell carcinoma, cervical cancer, and breast cancer. Endometrial cancer and colorectal cancer were excluded, as the primary indications for ICI in these sites are currently defined by microsatellite instability-high (MSI-H) molecular status (as of 8/2023). As MSI-H status was not available, a comparable historical control cohort could not be constructed. We excluded in-situ or non-invasive histologies when histology data was available from the Cancer Registry System. If there were multiple cancer diagnoses, the cancer with the most advanced stage at diagnosis was used; if there were multiple cancers of the same stage at diagnosis, the most recent diagnosis was used. For presentation purposes, the least common cancer types (gastric, breast, lymphoma, cutaneous SCC, Merkel cell, and cervix) were grouped as “Other” cancer type. Patients with missing covariate values (n=65, 0.2%) were excluded.

With this methodology, 72% of patients were successfully assigned a primary cancer type. For the remaining patients, we developed an ICD-9/10 code-based algorithm to assign primary cancer type. We identified all inpatient and outpatient cancer diagnosis codes corresponding to cancer types with approved ICI indications occurring within +/- 28 days of the index date. The cancer type with the greatest number of ICD codes in this window was then assigned as the primary cancer type. As ICD codes do not distinguish between non-small-cell lung cancer and small-cell lung cancer, we assigned SCLC as the primary cancer type if the patient had received atezolizumab or durvalumab along with etoposide and a platinum agent. Together, these methods successfully assigned cancer type to 95% of patients. Among patients with both Cancer Registry System data and ICD code cancer type assignment (n=19,639), the above algorithm resulted in a 90.4% agreement rate between the two methods (Cohen’s kappa 0.87, almost perfect agreement).

*Historical control group derivation.*

We developed a historical control cohort comprising patients with the same cancer types as the ICI cohort who were treated with conventional chemotherapeutics or targeted therapies and were never exposed to ICI (see below for list of included therapeutics). Patients were included if the systemic therapy start date was within 10 years before the first FDA approval of ICI for any indication in each cancer type. To provide adequate controls for the minority of ICI-treated patients who received adjuvant ICI in a setting where adjuvant systemic therapy would not have been commonly given in the pre-ICI era (e.g. adjuvant durvalumab after definitive chemoradiation for unresectable stage III NSCLC), we identified additional control patients in the Cancer Registry System who would have been eligible for adjuvant ICI; see Supplemental Table 3 for a detailed description of these criteria. For the adjuvant therapy control cohort, we included patients treated within 10 years before FDA approval of the specific adjuvant ICI indication.

For each control patient, we then identified start dates for each line of systemic therapy; see below for definition of lines of systemic therapy. For the control patients added to account for adjuvant ICI indications, the index date was defined as 6 weeks after the date of definitive surgery or radiotherapy completion as this is a typical timeframe in which adjuvant ICI would be initiated.^1^ To prevent an individual control patient from appearing multiple times in the analysis for each line of systemic therapy, we randomly selected one line among the second or later lines to use as the index date. The second or later line was used to improve matching performance to ICI patients, who preferentially received ICI in later lines of therapy. Using this assigned index date, we then assigned primary cancer type and diagnosis date using the same methodology as for the ICI cohort. Concordance between Cancer Registry System and ICD code cancer types was similarly high (n=53,381 patients; percent agreement = 90.6%; Cohen’s kappa 0.88). We excluded in-situ or non-invasive histologies when histology data was available from the Cancer Registry System. Patients with missing covariate values (n=357, 0.5%) were excluded. We further excluded patients undergoing systemic therapy regimens that were not concordant with treatment of invasive disease for their cancer type (n=7,454, 9%). To define concordant systemic therapies, we used National Comprehensive Cancer Network guidelines (accessed 8/14/23) to identify individual drugs or combination regimens with any current or historical indications in each cancer type. We also excluded patients with urothelial cancer of unknown stage at diagnosis undergoing single-agent mitomycin C, presumed to represent adjuvant therapy for non-invasive disease.

*Definition of lines of systemic therapy*.

For each patient, the first line of systemic therapy was defined as the first systemic therapy (targeted therapy, ICI, or chemotherapy) delivered after the cancer diagnosis date but before the index date. All drugs administered within 28 days of the first systemic therapy administration date were considered part of the first line of therapy. We then defined subsequent lines of therapy as the initiation of a new systemic therapy agent that was not included in any prior lines of therapy. For each new line of therapy, we determined the start date and included any systemic therapies administered within 28 days after the start date.

*Systemic therapies.*

CPT codes for included ICI drugs are shown in Supplemental Table 1. CPT codes for conventional systemic therapies are shown in Supplemental Table 2. Oral conventional chemotherapeutics included topotecan, melphalan, etoposide, capecitabine, and temozolomide. Methotrexate was excluded as oral methotrexate is primarily used for non-oncologic indications in the VA system. Targeted agents were drawn from all targeted therapies with FDA approval in each cancer type as of 8/14/23 and included enfortumab, erdafitinib, sacitizumab, abemaciclib, alpelisib, anastrozole, elacestrant, everolimus, exemestane, fulvestrant, lapatinib, letrozole, margetuximab, neratinib, olaparib, palbociclib, pertuzumab, ribociclib, sacituzumab, talazoparib, tamoxifen, toremifene, trastuzumab, tucatinib, bevacizumab, tisotumab, aflibercept, cetuximab, encorafenib, panitumumab, ramucirumab, regorafenib, tucatinib, axitinib, belzutifan, cabozantinib, everolimus, lenvatinib, pazopanib, sorafenib, sunitinib, temsirolimus, tivozanib, futabatinib, infigratinib, ivosidenib, pemigratinib, ramucirumab, regorafenib, sorafenib, brentuximab, binimetinib, cobimetinib, dabrafenib, encorafenib, tebentafusp, trametinib, vemurafenib, retifanlimab, adagrasib, afatinib, alectinib, amivantamab, bevacizumab, brigatinib, capmatinib, ceritinib, crizotinib, dabrafenib, dacomitinib, entrectinib, erlotinib, gefitinib, lorlatinib, mobocertinib, necitumumab, osimertinib, pralsetinib, selpercatinib, sotorasib, tepotinib, and trametinib.

**Supplemental Table 1.** CPT codes for ICI.

| **ICI** | **CPT code** |
| --- | --- |
| Pembrolizumab | C9027, J9271 |
| Nivolumab | C9453, J9299 |
| Atezolizumab | C9483, J9022 |
| Avelumab | C9491, J9023 |
| Durvalumab | C9492, J9173 |
| Tremelilumab | C9147, J9347 |
| Dostarlimab | J9272 |
| Cemiplimab-rwlc | J9119 |
| Ipililumab | J9228 |
| Nivolumab/relatlimab-rmbw | J9298 |

Abbreviations: CPT: Common Procedural Terminology

**Supplemental Table 2.** CPT codes for conventional chemotherapeutics.

| **Drug** | **CPT codes** |
| --- | --- |
| Capecitabine | J8520, J8521 |
| Cyclophosphamide | J8530, J9070, J9071 |
| Etoposide | J8560, J9181 |
| Gefitinib | J8565 |
| Melphalan | J8600, J9245, J9246, J9247 |
| Methotrexate | J8610, J9250, J9260 |
| Temozolomide | J8700, J9328 |
| Topotecan | J8705, J9351 |
| Doxorubicine | J9000 |
| Bendamustine | J9036, J9056, J9058, J9059 |
| Bleomycine | J9040 |
| Cabazitaxel | J9043 |
| Carboplatin | J9045 |
| Carmustine | J9050 |
| Cisplatin | J9060 |
| Cytarabine | J9098, J9100 |
| Dactinomycin | J9120 |
| Dacarbazine | J9130 |
| Daunorubicin | J9150, J9151, J9153 |
| Docetaxel | J9171 |
| Epirubicin | J9178 |
| Eribulin | J9179 |
| Fludarabine | J9185 |
| 5-fluorouracil | J9190 |
| Gemcitabine | J9196, J9198, J9199, J9201 |
| Irinotecan | J9205, J9206 |
| Ifosfamide | J9208 |
| Idarubicin | J9211 |
| Interferon | J9212, J9213, J9214, J9215, J9216 |
| Lurbinectedin | J9223 |
| Paclitaxel | J9259, J9264, J9265, J9267 |
| Oxaliplatin | J9263 |
| Mitomycin | J9280 |
| Pemetrexed | J9294, J9296, J9297, J9304, J9305, J9314, J9322, J9323 |
| Thiotepa | J9340 |
| Trabectedin | J9352 |
| Valrubicin | J9357 |
| Vinblastine | J9360 |
| Vincristine | J9370, J9371 |
| Vinorelbine | J9390 |

Abbreviations: CPT: Common Procedural Terminology

**Supplemental Table 3.** Criteria for inclusion of historical control patients for adjuvant ICI indications.

| **Site** | **ICI indication** |
| --- | --- |
| Melanoma | Adjuvant after complete resection of pathologic stage IIB, IIC, or III |
| Clear cell RCC | Adjuvant after surgery and either 1) grade 4 and stage II, or 2) stage III |
| Ureteral or renal pelvis, urothelial | Adjuvant after complete resection and pathologic stage T3 or T4 |
| NSCLC | Adjuvant after definitive chemoradiation for unresectable stage III |
| NSCLC | Adjuvant after complete resection and postoperative chemotherapy for locoregionally advanced disease |
| Urothelial bladder | Adjuvant after resection and neoadjuvant or adjuvant chemotherapy in muscle-invasive bladder cancer |
| Esophagus | Adjuvant after neoadjuvant chemoradiation and resection with residual disease for stage II-III esophageal or gastroesophageal junction cancer |

Abbreviations: RCC: renal cell carcinoma; NSCLC: non-small-cell lung cancer

**References**

1. Spigel DR, Faivre-Finn C, Gray JE, et al. Five-Year Survival Outcomes From the PACIFIC Trial: Durvalumab After Chemoradiotherapy in Stage III Non-Small-Cell Lung Cancer. J Clin Oncol 2022;40(12):1301–11.
